# The value of mNGS in the diagnosis of central nervous system infections in immunodeficient hosts with decompensated cirrhosis complicated by *Listeria encephalitis*: Case Report

**DOI:** 10.3389/fmed.2026.1857949

**Published:** 2026-07-20

**Authors:** Linman Cao, Yi Zhao, Rongrong Wang, Yijing Liu, Linzheng Luo, Han Yan, Na Li

**Affiliations:** 1 Graduate School of Hebei Medical University, Shijiazhuang, Hebei, China; 2 Hebei Provincial People's Hospital, Shijiazhuang, Hebei, China; 3 Graduate School of Hebei North University, Zhangjiakou, Hebei, China; 4 Graduate School of North China University of Technology, Tangshan, Hebei, China; 5 Key Laboratory of Brain Networks and Cognitive Impairment Diseases, Shijiazhuang, Hebei, China

**Keywords:** central nervous system infectious diseases, decompensated cirrhosis, immunodeficiency, *Listeria encephalitis*, mNGS

## Abstract

**Introduction:**

The incidence of central nervous system (CNS) infections caused by *Listeria monocytogenes* is rising, yet it remains rarely reported and frequently misdiagnosed in patients with decompensated cirrhosis. This report evaluates the diagnostic utility of metagenomic next-generation sequencing (mNGS) in this specific population.

**Case presentation:**

A 62-year-old male with a 7-year history of cirrhosis presented with fever, headache, and loss of consciousness. At admission, the patient was in a decompensated state with a Child-Pugh score of 9 (Grade B) and a Model for End-Stage Liver Disease (MELD) score of 12, characterized by hypoalbuminemia and mild ascites.

**Diagnosis and intervention:**

To avoid delayed treatment, broad-spectrum antibiotics were used before the results of blood and cerebrospinal fluid cultures were available. Preliminary cerebrospinal fluid (CSF) analysis showed an atypical inflammatory response in the context of cirrhosis-associated immune dysfunction. Although conventional CSF cultures remained negative, mNGS detected *Listeria monocytogenes* sequences within 16 h. Early mNGS-guided targeted therapy, followed by multidisciplinary management under real-world drug availability constraints, was associated with significant clinical improvement and successful discharge.

**Conclusion:**

Cirrhosis-associated immune dysfunction (CAID) and hypersplenism can mask typical CSF diagnostic markers. mNGS provides a rapid, unbiased diagnostic paradigm that is crucial for shortening diagnostic duration and guiding precision therapy in immunocompromised hosts.

## 1 Introduction

The decompensated stage of cirrhosis is characterized by portal hypertension and severe decline in liver function. When accompanied by complications such as ascites, gastrointestinal bleeding, and hepatic encephalopathy, it indicates that the liver has entered the decompensated stage (1). At this stage, liver function is severely impaired, manifested as decreased synthesis and detoxification functions and circulatory disorders. Patients at this stage have a comprehensive decline in immune defense function (2), making them prone to various infections, leading to septic shock, multiple organ failure, and even death.

*Listeria monocytogenes* (*LM*) is a Gram-positive facultative anaerobic bacterium that is widely distributed in nature (3) and can multiply in low-temperature environments. Infection with this bacterium is more common in people with weakened immune function (4) and can lead to central nervous system infection, bloodstream infection, pneumonia, etc. *Listeria* meningitis is a rare but critical central nervous system infectious disease (CNSIs) caused by *Listeria monocytogenes* (5). *LM* is an intracellular parasite that can penetrate the host’s intestinal barrier and enter the bloodstream, thereby infecting the central nervous system through the blood–brain barrier (6). The incubation period for neurolisteriosis is on average 10 days. Common symptoms include fever, headache, altered consciousness, and focal neurological deficits such as epilepsy and cranial nerve palsy (7). In recent years, the incidence of central nervous system infection caused by *Listeria* has increased, but central nervous system infection caused by *LM* in decompensated cirrhosis is rarely reported in clinical practice. This article reports a case of decompensated cirrhosis complicated with *Listeria encephalitis* admitted in April 2025, and discusses the clinical application value of mNGS in the early diagnosis of CNSIs in immunodeficient hosts in conjunction with the literature.

## 2 Case characteristics

### 2.1 Admission details

The patient was a 62-year-old male who was admitted to the hospital with the chief complaint of “fever, headache and loss of consciousness for 3 days.” No clear history of travel or animal contact in the past, and no consumption of unpasteurized or refrigerated food. Three days prior, he developed a fever without any obvious cause, with a maximum temperature of 38.0 °C, accompanied by pinprick pain in the scalp. The patient fell while using the toilet, lost consciousness, and had his eyes rolled back and his jaw clenched. The condition subsided on its own after about 1 min. He had a 7-year history of cirrhosis, the Child-Pugh score was 9 (Grade B) and the MELD score was 12, a history of diabetes and upper gastrointestinal bleeding. The patient’s cirrhosis was managed only with Biejia Jian Wan (a traditional Chinese medicine) without regular monitoring or antibiotic prophylaxis prior to admission.

Upon admission, the patient presented with altered mental status, dysarthria, and slowed response. The left upper limb muscle strength was grade 5, the right limb muscle strength was grade 3, and the right Babinski sign was positive. The abdomen resonated with tympanic percussion. Multiple spider angiomas were found on the forearm, arms, and both upper limbs. Superficial veins were tortuous and varicose around the umbilicus. Multiple purplish ecchymoses were found on the inner side of the right upper limb, the right side of the waist, and the outer side of the left femur.

Auxiliary examinations (Figures 1, 2): Head CT (Figures 1A–D) performed on hospital day 1 (HD1) revealed an asymmetric low-density area, which was interpreted as chronic ischemic changes or sulcal extension rather than acute infarction, ruling out new-onset stroke. Due to the unstable condition of the patient, we did not conduct a complete brain MRI. Abdominal CT (Figures 1E,F) scan performed on hospital day 1 (HD1) showed cirrhosis, splenomegaly, and ascites. Non-invasive intracranial pressure monitoring conducted on hospital day 1 (HD1) revealed the mean intracranial pressure was 204.40 mmH₂O. Cerebrospinal fluid examination results are shown in Table 1. Cerebrospinal fluid bacterial culture was generally negative.

**Figure 1:**
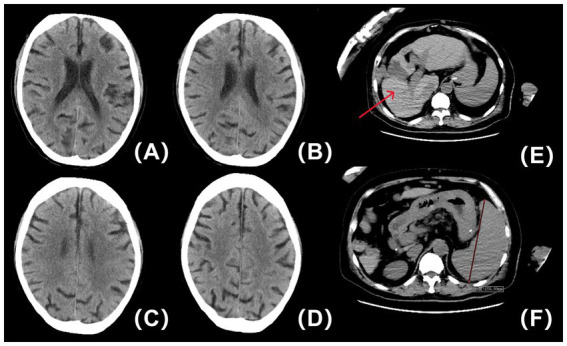
**(A–D)** Cranial CT shows an asymmetric low-density area, interpreted as chronic ischemic changes or sulcal extension, rather than acute infarction. **(E)** Decompensated cirrhosis liver CT: shows irregular liver contours, uneven liver density, and scattered high-density nodules. **(F)** Spleen CT scan: maximum diameter >12 cm, diagnosed as splenomegaly.

**Figure 2:**
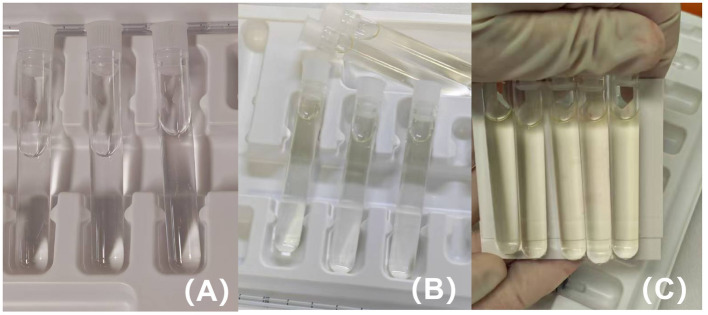
Cerebrospinal fluid: three cerebrospinal fluid images displayed in chronological order are labeled as **(A–C)**.

**Table 1:** Results of cerebrospinal fluid analysis from three lumbar punctures in the patient.

Detection time	Cerebrospinal fluid characteristics	CSF white blood cell count (×10^6^/L)	CSF neutrophils (×10^6^/L)	CSF lymphocytes (×10^6^/L)	CSF red blood cell count (×10^6^/ L)	CSF protein (mg/dL)	CSF glucose (mmol /L)	CSF chloride (mmol /L)	Cellular science (CSFC)
Admission (HD1)	Colorless and slightly cloudy	823	639	184	0	164.96	3.71	136.1	Predominantly neutrophils
First follow-up (HD4)	pale yellow and clear	88	18	70	0	107.75	5.54	138.1	Lymphocyte response is the main factor
Second follow-up (HD5)	Yellowish and slightly cloudy	82	10	72	3	99.40	6.22	138.6	Not submitted for testing

The results of metagenomic sequencing (mNGS) of pathogenic microorganisms (8) (Sampling on HD1, turnaround time in the lab is 16 h, and the report is issued on HD3): The CSF metagenomic next-generation sequencing (mNGS) ultra-sensitive double-testing (DNA & RNA) generated a total of 34,425,999 raw reads with a Q30 of 96.97%. After filtering host sequences, 735,839 non-human reads were retained. The assay successfully identified 3 unique reads belonging to the genus *Listeria* (relative abundance: 3.061%), with 2 reads specifically matching *Listeria monocytogenes*. Background microecology profiling also detected *Cutibacterium acnes* (44 reads) and *Staphylococcus warneri* (3 reads), which were identified as standard skin-flora contaminants from the lumbar puncture procedure rather than pathogens. Given the low absolute biomass, specific genome coverage data were inherently limited and approached zero. However, under a completely clean parallel negative control (NTC) and multi-factorial interpretative thresholds that prioritize pathogen pathogenicity over rigid numerical cutoffs, this low-read detection was clinically verified as a true positive.

### 2.2 Brief analysis and diagnostic process

The patient presented with unclear consciousness and fever of unknown origin, and experienced epileptic seizures prior to admission. Neurological examination revealed weakness in the right upper extremity, positive right Babinski sign, and negative meningeal irritation sign. The patient had a past medical history of liver cirrhosis, with multiple clinical manifestations including splenomegaly, ascites and coagulopathy. In accordance with the qualitative diagnostic principles of neurology, we consider that inflammatory disease is the most probable diagnosis. We hypothesize that the positive signs in this patient are caused by post-ictal Todd’s paresis combined with central nervous system inflammatory injury that damages the motor cortex and corticospinal tract of the brain, which further leads to pyramidal tract signs and hemiplegia. Cranial imaging examinations showed no acute focal ischemic lesions corresponding to the symptomatic region, therefore acute cerebral infarction is excluded.

As the cause of the illness could not be determined upon admission, empirical antibiotic therapy was initiated after collecting blood and cerebrospinal fluid samples. On hospital day 1 (HD 1), empirical antimicrobial therapy with intravenous ceftriaxone (4 g daily) and acyclovir (0.5 g q8h) was initiated upon admission. On HD 3, following the mNGS report confirming *Listeria monocytogenes* infection, acyclovir was discontinued, and targeted *anti-Listeria* therapy with ampicillin (3 g q6h) was promptly commenced. Notably, ceftriaxone was deliberately maintained alongside ampicillin. This combination was clinically indicated because the patient had sustained a femoral fracture during a loss-of-consciousness episode prior to admission, and an urgent surgical intervention was scheduled for HD 5, necessitating ceftriaxone as appropriate perioperative antibiotic prophylaxis. From HD 7 onwards, a severe and persistent regional supply shortage rendered ampicillin entirely unavailable. Faced with this real-world constraint, ampicillin had to be discontinued. Because of ampicillin unavailability, optimal *anti-Listeria* therapy could not be completed; the patient improved clinically after early targeted ampicillin exposure and supportive multidisciplinary care, but this case should not be interpreted as evidence that short-course ampicillin or ceftriaxone continuation is adequate therapy for *Listeria* CNS infection. Rather than interrupting all antibiotic treatment, ceftriaxone was pragmatically maintained until discharge on HD 17. The reason for this clinical decision is that the patient has been bedridden for a long time after a femur fracture surgery and developed a secondary lung infection, so continuing to use ceftriaxone can provide the necessary broad-spectrum antibiotic coverage.

Additionally, to prevent further deterioration of neurological function after outpatient seizure episodes, lacosamide (50 mg, twice daily) was empirically initiated. No clinical seizures were observed thereafter. Since there were no recurring seizure activities and the patient’s condition was critical, requiring other urgent interventions, electroencephalography (EEG) was not performed.

After treatment, the patient’s level of consciousness and limb weakness symptoms improved compared to before, the Babinski sign turned negative, body temperature decreased, no seizure recurrence was observed, the area of ecchymosis on the body had reduced compared to before, and cerebrospinal fluid indicators also improved. The patient was discharged on the 17th day of hospitalization.

### 2.3 Limitations of diagnosis and treatment

We did not perform a brain MRI. Indeed, magnetic resonance imaging of the brain is more sensitive for detecting parenchymal lesions, brainstem encephalitis, and microabscesses in *Listeria* infection, but in patients who are critically ill, with severe consciousness disorders, agitation, or unstable vital signs, its use is not always feasible. Cranial computed tomography remains the preferred emergency imaging method in the neurocritical care setting, used for quickly ruling out hemorrhage, mass lesions, and brain herniation. However, in this case, because MRI was not performed, the exact location of *Listeria* invasion in the brain parenchyma or meninges remains controversial, although this does not affect the etiological diagnosis and treatment established through metagenomic sequencing. Another limitation is the lack of electroencephalogram monitoring for this patient. Given the patient’s focal neurological deficits and history of seizures, an EEG could have provided objective evidence to distinguish Todd’s paralysis from nonconvulsive status epilepticus. However, after initiating treatment with lacosamide and ampicillin, the patient’s muscle strength and consciousness improved significantly, indicating that any potential seizures were successfully controlled, and the focal deficits may have been transient.

Additionally, the temporary alteration of the optimal *anti-Listeria* regimen due to medication unavailability represents a limitation inherent to real-world clinical practice, though it underscores the necessity of flexible, multidisciplinary management.

## 3 Discussion

### 3.1 Synergistic pathogenic mechanism of decompensated cirrhosis and *Listeria encephalitis*

Patients with decompensated cirrhosis exhibit significant cirrhosis-associated immune dysfunction (CAID). As shown in Figure 3, the core characteristic of CAID is the coexistence of congenital and acquired immune impairment with systemic chronic inflammation (9, 10). First, regarding the host susceptibility basis, this patient had a Child-Pugh score of 9 (Grade B) and a MELD score of 12, indicating a severe reduction in the liver’s ability to synthesize complement (e.g., insufficient C3/C4) and opsonins. Simultaneously, hypersplenism led to a decrease in the number of CD4 + and CD8 + T cells in peripheral blood and a weakened phagocytic capacity of monocytes/macrophages. In addition, the patient did not undergo antibiotic prophylaxis or systemic monitoring. This deep cellular immune deficiency, coupled with bacterial translocation caused by damage to the intestinal mucosal barrier, significantly reduces the body’s ability to clear intracellular pathogens such as *Listeria monocytogenes (LM)*. Secondly, regarding the mechanisms of bacterial invasion and central nervous system damage, *LM* is ingested orally and colonizes the intestines, using a “Trojan horse” mechanism (infecting monocytes) to directly invade the cerebral vascular endothelium and choroid plexus epithelium to cross the blood–brain barrier (11–13). After entering the central nervous system, *LM* multiplies extensively in the brain parenchyma, causing focal necrosis of brain tissue, microabscess formation, and inducing the massive release of pro-inflammatory factors such as TNF-*α*, IL-6, and IL-1β, ultimately evolving into severe *Listeria encephalitis*, and even leading to brain herniation and multiple organ failure. Finally, the CSF findings were atypical in the context of cirrhosis-associated immune dysfunction. During targeted treatment, this pattern gradually changed and improved over time. This atypical presentation is very deceptive and can easily lead to misdiagnosis in clinical practice (14).

**Figure 3:**
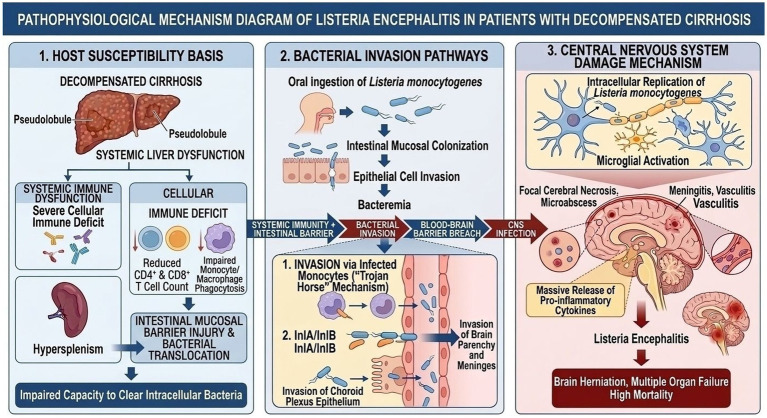
Pathological mechanism of *Listeria encephalitis* in decompensated cirrhosis: this model demonstrates the susceptible background (complement deficiency and T cell depletion), invasion pathways (Trojan horse mechanism), and resulting central nervous system damage and cytokine storm.

### 3.2 Innovation in the diagnostic model of intracranial infection with negative traditional tests

In this case, the early CSF routine and culture results failed to identify the pathogen, but the mNGS technology detected Listeria-specific sequences within 16 h, demonstrating its value in diagnosing rare, difficult-to-culture, and routinely negative CNSIs (15)(Figure 4). While FilmArray ME includes *L. monocytogenes*, mNGS provided superior diagnostic breadth for this complex patient by enabling unbiased screening for diverse opportunistic pathogens and co-infections not covered by fixed PCR panels. Especially for immunodeficient hosts, their CSF inflammatory markers are often atypical (16), and the diagnostic sensitivity of traditional methods is limited. A large-scale 2024 study in Nature Medicine (4,828 CSF samples) confirmed that mNGS had a sensitivity of 63.1% and detected 21.8% of pathogens missed by conventional methods, including *Listeria monocytogenes*, particularly in immunocompromised patients (17). Another 2024 study further validated that mNGS achieves significantly higher diagnostic yield than cultures in suspected CNS infections with atypical CSF profiles (18). In addition, in cases where cerebrospinal fluid cultures are negative, because *Listeria monocytogenes* is an obligate pathogenic intracellular bacterium rather than a common environmental or skin contaminant, even a low number of reads has clear diagnostic value when background noise is strictly controlled. This is fully consistent with the ‘Chinese Expert Consensus on the Diagnosis and Treatment of Central Nervous System *Listeria Monocytogenes* Infection,’ which proposes that in culture-negative cases, a positive cerebrospinal fluid mNGS result alone can serve as an independent and definitive etiological diagnostic criterion, and this has reached a 97% consensus among experts. Therefore, the mNGS can significantly improve the early diagnosis rate by detecting pathogen nucleic acids without bias (19), guide precise anti-infective treatment, and provide a new paradigm for the diagnosis and treatment of neuroinfections with “atypical changes” in cerebrospinal fluid. Nevertheless, to avoid the overinterpretation of low-biomass results, the definitive etiological diagnosis in our specific case was established based on the comprehensive integration of the patient’s clinical presentation, atypical cerebrospinal fluid findings, host-specific risk factors, a clean parallel negative control, the absence of alternative pathogens, and the clinical response to early therapy, rather than relying on the low number of mNGS reads alone.

**Figure 4:**
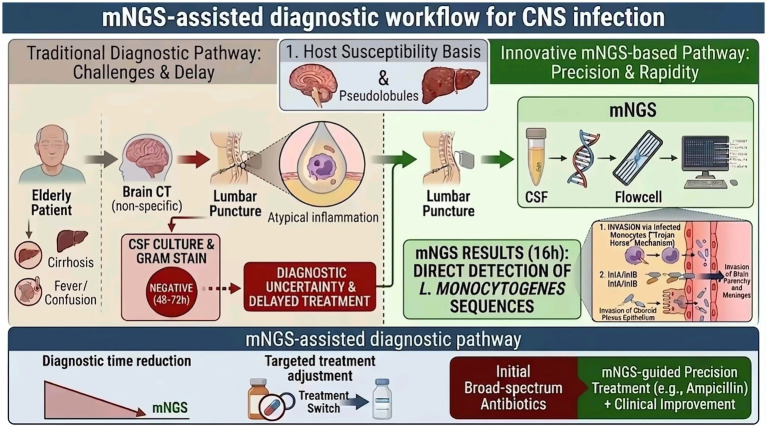
Clinical diagnostic flowchart emphasizing the impact of mNGS: a comparative paradigm showing how the mNGS-based pathway reduces diagnostic time from days to hours and shifts management from initial broad-spectrum antibiotics to targeted precision therapy.

### 3.3 Clinical implications

The clinical significance of this study lies in confirming a successful ‘mNGS-to-targeted-therapy’ loop. Unlike fixed PCR panels, unbiased mNGS ruled out complex co-infections in this vulnerable patient, and its rapid 16-h turnaround enabled early targeted anti-Listeria therapy, though the recommended full treatment course could not be completed owing to unexpected real-world ampicillin unavailability.

For patients with immunodeficiency such as cirrhosis, the appearance of abnormal neurological symptoms should raise suspicion of opportunistic infections such as *Listeria*, prompting early mNGS testing to shorten the diagnostic time. Treatment requires multidisciplinary collaboration, including antimicrobial therapy, active immune modulation, liver function support, and prevention and treatment of complications.

Further prospective studies are needed to determine the optimal timing of mNGS application in patients with cirrhosis and CNSIs, and to explore the role of immune reconstitution strategies in improving prognosis.

## Data Availability

The original contributions presented in the study are included in the article/supplementary material, further inquiries can be directed to the corresponding author.
